# A Parietal Biomarker for ADHD Liability: As Predicted by the Distributed Effects Perspective Model of ADHD

**DOI:** 10.3389/fpsyt.2015.00063

**Published:** 2015-05-07

**Authors:** T. Sigi Hale, Joshua F. Wiley, Susan L. Smalley, Kelly L. Tung, Olivia Kaminsky, James J. McGough, Ashwin M. Jaini, Sandra K. Loo

**Affiliations:** ^1^Department of Psychiatry and Biobehavioral Sciences, University of California Los Angeles Semel Institute for Neuroscience and Human Behavior, Los Angeles, CA, USA; ^2^Department of Psychology, University of California Los Angeles, Los Angeles, CA, USA

**Keywords:** attention, ADHD, laterality, asymmetry, parietal, risk factors, liability, DRD4

## Abstract

**Background:**

We previously hypothesized that poor task-directed sensory information processing should be indexed by increased weighting of right hemisphere (RH) biased attention and visuo-perceptual brain functions during task operations and have demonstrated this phenotype in ADHD across multiple studies, using multiple methodologies. However, in our recent distributed effects model of ADHD, we surmised that this phenotype is not ADHD specific, but rather more broadly reflective of any circumstance that disrupts the induction and maintenance of an emergent task-directed neural architecture. Under this view, increased weighting of RH-biased attention and visuo-perceptual brain functions is expected to generally index neurocognitive sets that are not optimized for task-directed thought and action, and when durable expressed, liability for ADHD.

**Method:**

The current study tested this view by examining whether previously identified rightward parietal EEG asymmetry in ADHD was associated with common ADHD characteristics and comorbidities [i.e., ADHD risk factors (RFs)].

**Results:**

Barring one exception (non-right handedness), we found that it was. Rightward parietal asymmetry (RPA) was associated with carrying the DRD4-7R risk allele, being male, having mood disorder, and having anxiety disorder. However, differences in the specific expression of RPA were observed, which are discussed in relation to possible unique mechanisms underlying ADHD liability in different ADHD RFs.

**Conclusion:**

Rightward parietal asymmetry appears to be a durable feature of ADHD liability, as predicted by the Distributed Effects Perspective Model of ADHD. Moreover, variability in the expression of this phenotype may shed light on different sources of ADHD liability.

## Introduction

We recently presented a distributed effects perspective (DEP) model of ADHD that addresses specific challenges associated with the elucidation and treatment of dimensionally defined psychiatric disorders (title: A Distributed Effects Perspective of Dimensionally Defined Psychiatric Disorders: And Convergent versus Core Deficit Effects in ADHD) (1). The model presents a novel approach to conceptualizing brain-function pathology in dimensionally defined disorders (e.g., distributed versus convergent deficit effects) and makes several predictions regarding abnormal brain function in ADHD. The current study tests one of its key predictions. Below, we present a brief summary description of: (1) the background conceptual framework, (2) research underlying the models genesis, (3) a cursory description of the model, and (4) the rationale for the current empiric study. The overarching goal of this work is to test and refine the DEP model of ADHD, and as such, we present and discuss this study in that context.

### The conceptual framework

Right hemisphere (RH) specialization is well established for top–down task-directed attention functions, such as vigilance, sustained, and selective attention (2–7). It is also reported for bottom–up functions, such as: detection of sequence breaking novel objects (8), automatic assessment of object relevance (9), automatic perceptual-integrative category learning (10, 11), esthetic analysis (12), and within-category feature discrimination (13). Within these top–down and bottom–up domains, we can further distinguish RH processing that supports fast identifications versus in-depth sensory analysis of stimuli. For instance, top–down processing can facilitate quick stimulus identification using the minimal sensory detail required (i.e., effortful categorizations), or the effortful scrutiny of stimulus details (14). Likewise, bottom–up processing can facilitate fast automatic detection of behaviorally relevant content in our surroundings (i.e., automatic categorizations), or fluid/unguided sensory-immersive experience (15, 16). In short, multiple forms of RH contribution to visual processing can be distinguished; in our work, we have emphasized four specific aspects: (1) top–down task-directed categorizations, (2) top–down task-directed scrutiny of details, (3) bottom–up automatic categorizations, and (4) bottom–up sensory-immersive.

The current line of research began with the precept that complex task-directed actions heavily rely on the first of these (i.e., making fast top–down categorizations of stimuli, using the minimal sensory detail required). We conceptualized this as “task-specialized” sensory information processing, and considered it to reflect variable mixtures of selective, sustained, and/or vigilance related functions depending on the nature of a given task. We hypothesized that ADHD involves a specific deficit for, and/or reduced expression of this task-specialized manner of sensory processing, with a proportional increased expression of non-task-specialized forms (items 2–4 above). We posited that this circumstance should result in mixed expression of: (a) unneeded scrutiny of visual details, (b) increased attentional shifting to off-task content, and/or (c) task-inappropriate orientation toward sensory-immersive processing. The net effect being an increased attentional effort and exposure to visual sensory content beyond what is strictly required to perform task operations. We conceptualized this as “visual sensory overflow” (in relation to task objectives), and predicted that it would be indexed by a relative increased weighting of visuo-perceptual versus verbal-categorical processing during task challenges.

### Supporting literature

We have performed and published three behavioral laterality (17–19), two fMRI (20, 21), and four EEG (22–25), studies examining this view. All produced convergent findings in support of increased RH contribution to sensory processing in ADHD. The behavioral laterality studies showed greater RH contribution to processing task-stimuli, associated left hemisphere (LH) linguistic deficits, and abnormal interhemispheric interaction (17–19). This work also suggested that RH-biased processing in ADHD reflects abnormal brain-state orientation (rather than capacity limitations) (19), bears advantages for RH specialized signal detections (negative tone of voice) (19), impacts high-order cognition (17), and is linked to a superior capacity to inhibit prepotent LH responsivity (19). The fMRI and EEG studies demonstrated that RH bias in ADHD is mainly evident during sub-executive operations (20), exhibits stronger expression with greater ADHD family loading (23), and is identified in visual (21), inferior and temporal-parietal (22, 25), and frontal (24) brain regions. Moreover, a robust and literature-consistent (26, 27) biomarker was identified. ADHD subjects exhibited pronounced rightward EEG beta asymmetry (16–21 Hz) in inferior parietal brain regions during the Conner’s continuous performance test (CPT) (25). We have replicated this finding (22), and found it to also be present in temporal-parietal regions, along with greater unilateral right-sided beta power in the superior parietal lobe (SPL) (22). This study also replicated an effect showing abnormally weak association between inferior and temporal-parietal beta asymmetry in ADHD. Finally, our most recent fMRI study identified rightward biased visual-cortical function in ADHD, and showed that this characteristic is linked to multiple distributed network systems, including the default mode and the right-lateralized ventral attention networks (21).

Although not yet widely understood, the above findings are well aligned with extant ADHD literature. Abnormal information processing in ADHD is now well established (28, 29), with slow naming speed identified (30–37). Previous behavioral laterality studies have indicated greater RH contribution (38, 39). Functional imaging studies at rest or during simple (i.e., sub-executive) challenges have shown a pattern of reduced LH (40–43) and/or increased RH contribution (20, 24, 44–46). Recent diffusion tensor imaging studies have reported greater RH parietal (47) and frontal (48) fractional anisotropy. A lack of normally occurring L > R asymmetry in prefrontal cortical convolution complexity has also been reported (49), as well as increased RH visual cortex volumes (50). Finally, abnormal posterior corpus callosum size (51) and function (45, 52–54), including abnormally fast left-to-right callosal transfer times (52), clearly implicate some form of abnormal integration of verbal and non-verbal sensory operations in ADHD.

With complex executive function (EF) tasks the literature is more variable, showing diffuse effects mainly consistent with variable weaknesses across multiple brain systems (55–58). Nevertheless, several studies have found an increased association between ADHD behavioral performance and right-sided brain structure and/or function (59–66), and EEG studies that directly examined activation asymmetry and/or left-right hemisphere differences have consistently shown a R > L pattern in posterior brain regions (23–25, 26, 27, 44, 45, 67). A recent meta-analysis of ADHD functional imaging also reported hyper-activation of the strongly right-lateralized ventral attention network (VAN), noting it may be related to increased bottom–up visuo-perceptual processing of task-extraneous stimuli (28), In this vein, Fassbender and Schweitzer (68), in an earlier review of ADHD brain imaging literature, suggested that ADHD involves an increased reliance on neuroanatomy associated with visual/spatial and motoric (versus verbal) processing.

Finally, increased RH relative to LH contribution may be consistent with common ADHD characteristics, such as: increased leftward motor preference (69, 70), which has been linked to greater RH contribution to language (71, 72); being male (73), as males tend to exhibit better visual-spatial and poorer linguistic abilities compared to females (74, 75), and dysregulated dopamine and noradrenergic function (76), as these systems appear to exhibit a degree of LH and RH specialization respectively (77).

In sum, abnormal information processing is now well established in ADHD. Our work, in association with the above literature, strongly suggests that this abnormality is linked to an atypical increased weighting of RH visuo-perceptual versus LH verbal-categorical processing.

### The DEP model of ADHD

The distributed effects perspective (DEP) model of ADHD asserts that task-directed brain functioning, including task-specialized sensory information processing, is facilitated by the induction and maintenance of an emergent neural architecture (neurocognitive network or brain state) that optimizes multiple distributed systems toward task-directed thought and action (78). The model refers to this emergent neural architecture as a task-directed adaptive processing state (TD-APS) and conceptualizes it to be comprised of four primary computational nodes: (1) verbal working memory (VWM) to sequence, direct, maintain, and update task directives (with possible support from spatial working memory to model integrated plan steps) (79–82), (2) spatial working memory (SWM) to generate predictive sensory models to help bias downstream processing toward task stimuli (83, 84), (3) fast perceptual identification of task-relevant content (14, 16), and (4) translation of perceptual content into verbal articulatory codes that can be readily integrated with, and used to update, task-plans in VWM (85). This system covers four essential task operations: planning, sensory modeling, perceptual encoding, and verbal encoding. It is conceived to have the primary internal objective of orchestrating task directives, and the primary external objective of asserting top–down task-directed control over sensory information processing. The induction and maintenance of this system is expected to coincide with the suppression of automatic sensory and motoric responsivity (86), and requires the dynamic coordination of multiple distributed network systems (e.g., default mode, dorsal and ventral attention, etc.) [For full model description, see Ref. (1)].

The DEP model suggests that ADHD symptom sequelae manifest from TD-APS system impairment, no matter the cause, and that ADHD heterogeneity reflects the diversity of ways in which any such complex distributed system may come to be compromised. Under this view, common ADHD characteristics and/or comorbidities are predicted to reflect common factors that imperil TD-APS system functioning (e.g., sleep-disorder, novelty seeking temperament, day-dreaming temperament, anxiety, mood, linguistic impairment, working memory impairment, slow processing speed, etc.). Furthermore, “pure ADHD” is conceived to reflect incidences where TD-APS system impairment is both life-disruptive and linked to subclinical causal impairments to its constituent elements. If such impairments are clinically manifest (e.g., impaired verbal encoding), an alternative primary and/or comorbid diagnosis is expected (e.g., dyslexia, slow processing speed, etc.).

An additional important assertion of the model is that variable causal deficits to the TD-APS system are expected to produce “convergent deficit effects” that reflect the system being less able to achieve its primary computational goals. Specifically, TD-APS system impairment, no matter the cause, is expected to result in: (1) a reduced capacity to orchestrate task directives (internal-convergent deficit), indexed by poor working memory and EF-control; (2) a reduced capacity to assert top–down task-directed control over sensory information processing (external-convergent deficit), indexed by increased weighting of RH-biased visuo-perceptual versus left hemisphere (LH) biased verbal-categorical processing; and (3) a general loss of TD-APS system stability (general-convergent deficit), indexed by greater performance variability.

Each of these deficit effects has been established in ADHD [for review, see Ref. (1, 87–91)]. However, the DEP model asserts that they are not ADHD specific, but rather that they are general indicators of poor TD-APS system functioning. If true, the proposed convergent outcomes should be generally present with any circumstance that is tied to poor task-directed brain functioning. Moreover, when such circumstances are time-extended and/or trait-link, they should impart risk for ADHD and be more frequently present in ADHD samples (i.e., common ADHD characteristics and comorbidities).

### The current study

The current study tests the assertion that previously identified RH-biased sensory information processing in ADHD is reflective of the DEP model’s proposed external-convergent deficit effect. If true, this characteristic (RH-biased processing) should be directly linked to common ADHD characteristics and comorbidities [i.e., ADHD risk factors (RFs)]. To test this hypothesis, we used a large previously collected sample of ADHD children to examine the impact of: (1) being male (73), (2) carrying at least one copy of the DRD4 7 repeat allele (92), (3) having comorbid mood disorder (93), (4) having comorbid anxiety disorder (93), and/or (5) having reduced right-sided motor dominance (69, 70) on rightward parietal EEG asymmetry, and whether this phenotype is associated with ADHD symptoms. As a secondary behavioral validation of RH-biased brain function, we also tested lateralized signal detection using a dichotic listening paradigm. This set of ADHD RFs reflects those that were identified in the literature to be associated with ADHD and that were present in sufficient numbers in our sample to justify analysis.

As a secondary objective, we also performed exploratory analyses to test whether individual ADHD RFs might be associated with different underlying causal impairments to the proposed TD-APS system. We reasoned that if ADHD RFs are associated with unique causal deficits to this system (distributed deficit effects), rightward parietal EEG asymmetry (the convergent outcome) should exhibit different patterns of association to metrics that tax TD-APS system constituent elements. For example, if the primary mechanism underlying TD-APS system weakness among males is reduced verbal ability relative to females, then male-related increases in rightward parietal EEG asymmetry should exhibit an association to verbal metrics. To explore this idea, for each ADHD RF, we examined the pattern of association between rightward parietal EEG asymmetry and a battery of temperament and cognitive metrics. We also conceptualized that different “points of deficit origination” across TD-APS brain-system constituent elements could result in variant expression of RH-biased brain function, as indexed by variability in the frequency, location, and testing-condition of rightward parietal EEG asymmetry in ADHD RFs.

## Materials and Methods

### Subjects

The sample consisted of 274 children from an earlier ADHD family genetics study (94, 95). Subjects came from 169 families and comprised of: 79 singletons, 77 sib-pairs, 11 trios, and two quads. After receiving verbal and written explanations of study requirements, a parent and the participating child provided written informed consent/assent, as approved by the UCLA Institutional Review Board. To screen for ADHD and other psychiatric disorders participating children and their mothers were interviewed using the semi-structured interview of the Kiddie Schedule for Affective Disorder and Schizophrenia for School-Age Children-Present and Lifetime Version (K-SADS-PL) (96). Autism was ruled out via the Social Communication Questionnaire (97). All interviews were conducted by clinical psychologists or highly trained interviewers with extensive experience in psychiatric diagnoses. “Best estimate” diagnoses were determined after individual review of diagnoses, symptoms, and impairment level by a board certified child psychiatrist (98). Inter-rater reliabilities were computed with a mean weighted kappa of 0.84 across all diagnoses with a greater than 5% occurrence in the sample. Subjects were excluded based on the following criteria: currently taking psychoactive medication, past or current documented neurological disorder, a significant head injury resulting in concussion, a diagnosis of schizophrenia or autism, or an estimated Full Scale IQ < 80. Inclusion criteria for the present study required a current diagnosis of ADHD. Subjects on stimulant medication were asked to discontinue use for 24 h prior to their visit.

#### ADHD RFs

Identification of subjects with comorbid mood was based on a definite diagnosis of at least one current mood disorder as assessed by direct interview using the Schedule for Affective Disorders and Schizophrenia-Lifetime Version (SADS-LAR). Identification of subjects with comorbid anxiety required definite diagnosis of at least two current anxiety symptoms as assessed by direct interview using SADS-LAR.

A DRD4 7R group was identified based on subjects possessing at least one copy of the seven-repeat allele (ref). Blood samples were collected from subjects, and DNA was isolated using the Puregene Kit following the manufacturer’s recommendations (Gentra Systems, Minneapolis, MN, USA). DRD4 polymorphisms (48-bp VNTR and 120-bp repeat) were genotyped according to standard procedures and have been reported elsewhere (99). Since the DRD4-7-repeat allele is thought to be the “risk” allele for ADHD, ADHD subjects were stratified according to whether they possessed at least one copy of the seven-repeat allele (7R) or not (not 7).

Handedness was assessed with a shortened version of the Edinburgh Handedness Inventory (100). This scale uses seven questions regarding hand preference and produces scores ranging from negative 14 (indicating maximum left-handedness), to positive 14 (indicating maximum right handedness). Due to insufficient sample size, we could not produce a purely left-handed RF group. Instead, scores from the Edinburg Handedness questionnaire were dichotomized so that scores ranging from 8 to 14 indicated “strong right handedness,” and scores less than 8 indicating “not definite right handedness.”

Using a score of less than two standard deviations below the mean on any or our standardized measures of reading, phonologic, or spelling ability produced a small sample (n = 16) comprised of variable linguistic weaknesses. Hence, we chose to not include reading disability as an ADHD associated RF in this study. There were no significant differences in linguistic ability based on RF status, as assessed by measures of reading, spelling, phonological, and vocabulary skill (see Table 2 for description of linguistic measures). Subject demographic information is presented in Table 1.

**Table 1:** **Sample demographics (*n* = 274)**.

Measures	Statistic
Age	$\bar{x}$ = 10.9, std = 3.4
Estimated IQ	$\bar{x}$ = 111.3, std = 15.5
ADHD Type	152 C, 113 I, 9 H
**ADHD risk factors**
DRD4 7R	88 with 7R allele
Males	177 Males
Mood	26 Affected
Anxiety	43 Affected
Not strong right handed	31

*Estimated full IQ: estimated from block design and vocabulary subtest of WAIS-R; ADHD type: C, combined; I, inattentive; H, hyperactive; Mood reflects definite diagnosis of at least 1 current mood disorder as assessed by direct interview using SADS-LAR (see text for reference). Anxiety reflects definite diagnosis of at least 2 current anxiety symptoms as assessed by direct interview using SADS-LAR (see text for reference)*.

### General testing procedures

Typical testing procedures for the UCLA ADHD genetics study involved a mother and/or father and two ADHD affected offspring coming to UCLA for a single visit (although fathers were often tested on a separate day). During the visit, each family member underwent a clinical, cognitive, and EEG testing battery, with the order of delivery of each component determined by logistical considerations. However, during EEG testing the Conners’ CPT (101) was delivered first, followed by additional conditions that are not reported. Recordings were performed in a small, private room with a sole male technician administering the protocol.

### EEG procedures

EEG recording was carried out using 40 silver chloride electrodes using the International 10/20 locations and was referenced to an average of singles recorded separately at each ear lobe. Eye movements were monitored by electrodes placed on the outer canthus of each eye for horizontal movements and by electrodes above the eye for vertical eye movements. EEG recording consisted of 2 baseline conditions lasting 5 min each [eyes open (EO) and eyes closed (EC)] and a cognitive activation condition lasting 15 min (Conners CPT-II) (102).

Continuous EEG data were subjected to automatic artifact detection via MANSCAN software (SAM Technology, Inc., San Francisco, CA, USA) designed to identify dead and bad channels, vertical and horizontal eye movements, saturation, muscle and movement artifact, and line frequency noise. Subsequent to this automated procedure an experienced EEG technician then visually inspected all data and identified any residual contaminants. Next, continuous EEG was broken into 1-s epochs and artifact-containing epochs were removed on a channel specific basis. Remaining artifact free epochs were then Fast Fourier Transformed (FFT) using MANSCAN EEG software, which uses a Welch’s Periodogram approach (103). We specified 1-s data segments, with 50% overlap, and a Hanning Windowing function to generate spectral content at a 1 Hz resolution. Spectral data were then averaged for each condition (EC, EO, CPT), and EEG power (mv^2^) from 1 to 21 Hz was exported in 1 Hz bins (e.g., 0–1, 1–2, … 20–21). Technicians involved in the EEG recording and processing were blind to ADHD diagnostic status.

For the current study, we evaluated the EEG frequency bands that were previously associated with abnormal EEG asymmetry in ADHD (23–25, 26, 27, 44, 67). Absolute power between 8–10 Hz, 10–12 Hz, 12–16 Hz, and 16–21 Hz frequencies were averaged for each electrode composing “low” and “high” alpha and beta measures, respectively (A1, A2, B1, B2). Our primary interest was to evaluate relative R > L parietal brain activation in RF groups. We therefore utilized measures of power asymmetry rather than examining group differences separately in each hemisphere. Laterality indices (LIs) were generated for three homologous right–left parietal electrode pairs (TP8-TP7, P4-P3, P8-P7) using the following standard calculation: [(R − L)/(R + L) * 1000]. Greater values for LIs mean greater rightward asymmetry. EEG asymmetry indices are notated as follows: condition (EC, EO, CPT), frequency (A2, A2, B1, B2), parietal region (TP8-7, P4-3, P8-7), for example, EC A1 TP8-7.

### Clinical and behavioral measures

Commonly used measures are listed in Table 2. Selected measures are also described here. Note: some tasks were added at different stages of the ADHD family genetics study, which is reflected by smaller sample sizes for some measures (e.g., CPT task).

**Table 2:** **Summary of behavioral measures**.

Instrument	Measures	Abbrev.	Reference
**ADHD symptoms**
Schedule for affective disorder and schizophrenia for school-age children-present and lifetime version (KSADS-PL)	Hyperactive/inattentive symptom counts	KSAD-H	(96)
		KSAD-I	
Swanson, Nolan, and Pelham-IV (SNAP-IV) parent rating scale	4 point Likert scale assessment of hyperactivity and inattention	SNAP-H	(148)
		SNAP-I	
**Temperament**
Child behavior checklist (CBCL) social problem scale, plus two recently identified factor items.	Social problems	Soc Prob	(104, 105)
	Social immaturity^a^	Soc Imm	
	Peer rejection^a^	Peer Rej	
The junior temperament and character inventory scale (JTCI)	Novelty seeking	NS	(136)
	Harm avoidance	HA	
	Reward dependence	RD	
	Persistence	PR	
	Self-directedness	SD	
	Cooperativeness	CP	
	Self-transcendence	ST	
**Cognition**
Wechsler intelligence scale for children, third edition (WISC-II)	Block design	Blocks	(149)
	Vocabulary	Vocab	
	Coding	Coding	
	Arithmetic	Arith	
	Digit span forward max	DSF-max	
	Digit span forward acc	DSF-acc	
	Digit span backward max	DSB-max	
	Digit span backward acc	DSB-acc	
	Spatial span forward max	SSF-max	
	Spatial span forward acc	SSF-acc	
	Spatial span backward max	SSB-max	
	Spatial span backward acc	SSB-acc	
Peabody individual achievement test-revised (PIAT-R)	Reading recognition	Read_rec	(150)
	Spelling	Spell	
Woodcock Johnson-revised, word attack subtest (WJ-R)	Phonologic processing	Phono	(151)
Stroop task	Color naming	St-Color	(152, 153)
	Word naming	St-Word	
	Interference	St-Inter	
The trail making test – A and B	Trails A	Trails A	(154)
	Trails B	Trails B	
Conner’s continuous performance test II (CPT-II)	Commissions	Commiss	(101)
	Omissions	Omiss	
	Hit reaction time	Hit RT	
	Hit RT standard error	Hit RTSE	
	Sensitivity	D-prime	
	Bias	Beta	
Spatial working memory (SWM)	Load 1 accuracy	SWM L1 Acc	(107)
	Load 1 reaction time	SWM L1 RT	
	Load 1 RT standard dev. (+ loads: 3, 5, 7)	SWM L1 RTSD (+ load: 3, 5,7)	
Dichotic listening emotion/words	Left-ear words (Acc/RT)	LE-word	(127)
	Right-ear words (Acc/RT)	RE-word	
	Left-ear emotion (Acc/RT)	LE-emot	
	Right-ear emotion (Acc/RT)	RE-emot	

*^a^Novel indices from CBCL derived from factor analysis (see text for reference)*.

#### Temperament

Social functioning characteristics were assessed using the mother’s report on the Social Problem scale of the child behavior checklist (CBCL) (104), as well as two previously identified factors from this scale (105). The first factor called “social immaturity” included items 1 (acts young), 11 (clings), 62 (clumsy), and 64 (prefer young). The second factor called “peer rejection” included items 25 (not get along), 48 (not liked), and 39 (teased). The Junior Temperament and Character Inventory scale (JTCI) was also used. This measures individual differences on seven personality dimensions: four dimensions of temperament (novelty seeking, harm avoidance, reward dependence, persistence), and three dimensions of character (self-directedness, cooperativeness, self-transcendence).

#### Conner’s Continuous Performance Test II

The CPT requires subjects to monitor a central fixation on a computer screen while single capital letters are sequentially and centrally presented during six continuous blocks of 20 trials with either 1, 2, or 4 s inter-stimulus intervals (ISIs) (two blocks for each ISI). The order of ISI-block presentation is randomized within subjects. The task requires subjects to press the space bar using their dominant hand with every letter presentation except for the letter “X.” The “X” occurs on 10% of the trials within a given ISI block. Behavioral performance was assessed using the following standard measures (106): (1) commission errors: a failure to inhibit response when an “x” is presented, (2) omission errors: a failure to respond when any letter other than “x is presented, (3) hit reaction time: response time for all letters other than “x,” (4) hit reaction time standard error: reaction time variability, (5) response bias: signal detection measure (beta) indicating impulsive versus conservative response styles, and (6) sensitivity: signal detection measure (d-prime) indicating accuracy adjusted for false alarms.

#### Spatial Working Memory

In this spatial delay response task (SDRT) (107), subjects were shown a target array of 1, 3, 5, or 7 yellow circles positioned pseudo-randomly around a fixation point. Then, after a fixed delay, they were shown a single green circle and had to indicate if it was in the same position as any of the previous yellow circles. Trial events included a 500-ms initial period of blank screen, 500 ms of a fixation point, a 2-s target array presentation, a 3-s delay period (with fixation), and a 3-s fixed response interval. Fifty percent of the trials were true positive, and 50% were true negative. Responses (yes or no) and reaction times were recorded for each trail. The number of correct responses, mean reaction time, and reaction time variability were recorded.

#### Dichotic Listening for Emotions and Words

This task requires subjects to detect an emotional tone of voice or to identify a word during dichotic presentations of four words (bower, dower, power, tower) spoken in four different emotional tones (happy, sad, angry, neutral) by a male speaker. Stimuli for the “word” and “emotion” tasks are identical and only the instructions to the subject change between conditions. Because dichotic presentations suppress ipsilateral auditory channels, the stimuli in each ear project to the opposite hemisphere via fibers that cross at the superior olive and inferior colliculus (108, 109). Thus, if each hemisphere can process the stimuli presented in the contralateral ear [direct access: (110)], then subjects’ ability to detect signal in the right ear, for instance, is indicative of left hemisphere (LH) competence. Based on previous work, we used nominated targets “bower” and “sad,” as these show the most robust LH and RH specialization, respectively. Moreover, with these stimuli, right-ear presentations of the “sad tone of voice” (projecting to the LH) are expected to require a left-to-right callosal transfer to the RH (111). Likewise, left-ear presentations of the word “bower” (projecting to the RH) are expected to involve right-to-left transfer to the LH, although some RH competence for the word signal is also expected (111). This task consistently demonstrates a left-ear (RH) advantage for processing emotional intonation and a right-ear (LH) advantage for processing words (112).

### Data analytic procedures and results presentation overview

We first present analyses to characterize ADHD RF groups with respect to clinical, temperament, and cognitive characteristic. Next, analyses aimed at testing the DEP model of ADHD are presented and discussed in two parts. Part 1 addresses our primary aim of examining whether RH bias is a general feature of ADHD RFs. Part 2 addresses our secondary/exploratory aim of examining whether ADHD RFs show different patterns of association between RH bias and temperament and/or cognitive characteristics. Section specific discussions are presented for Parts 1 and 2, followed by a general discussion. A summary of all study findings is provided in the supplement (Table S3 in Supplementary Material).

#### Non-Independence

Non-independence in our data (sibling pairs) is distributed across the levels of our grouping variables, making it somewhat ambiguous with respect to increasing our decreasing our ability to detect RF group differences. For instance, with regard to the “being male” RF, siblings are just as likely to be the same or different genders. If different, non-independence hurts our capacity to detect a gender difference – if the same, it helps. Moreover, because our emphasis was to uncover conceptually meaningful patterns, rather than individual effects, our interpretation of results is conceptually insulated against type 1 error. Nevertheless, steps were taken to reduce this risk (described below).

#### Multiple Testing

In primary analyses of rightward parietal asymmetry (RPA) in ADHD RFs (Part 1), the multivariate multilevel modeling approach limited the number of tests and accounted for non-independence (by modeling family membership). Analyses to characterize our samples, and secondary/exploratory analyses (Part 2), utilized large batteries of cognitive and temperament metrics (41 measures – see Table 3), and so were vulnerable to type 1 error. However, as noted, our goal in performing these analyses was to examine patterns of results among different RFs (i.e., we do not interpret individual findings). Nevertheless, to help guard against false positives diluting, distorting, or disrupting otherwise coherent patterns, and to reduce the complexity of this large set of findings, results for temperament and cognitive outcomes are restricted to a p threshold of 0.015. Symptom metrics included only four measures, and because all subjects had ADHD, we expected limited variability in these measures. Hence, we did not restrict symptom findings to a 0.015 threshold.

**Table 3:** **General characteristics by ADHD risk factor type**.

RFs and measures	No RF		With RF		Statistics			With RF
	$\bar{x}$	se	$\bar{x}$	se	df	*f*	*P*-value	
**Symptoms** (handedness no effects)								
*DRD4*								
KSAD-I	8.1	(0.13)	7.5	(0.19)	1,104	6.8	0.010	Less
*Sex*								
KSAD-H	5.0	(0.26)	5.7	(0.21)	1,260	5.2	0.024	More
SNAP-H	1.53	(0.07)	1.76	(0.06)	1,255	6.4	0.012	More
*Mood*								
KSAD-H	5.3	(0.17)	6.7	(0.57)	1,260	5.6	0.019	More
*Anxiety*	
KSAD-H	5.3	(0.18)	6.2	(0.43)	1,260	3.8	0.053	More
**Temperament** (*P* threshold. 015: DRD4 7R, handedness no effects)								
*Sex*								
RD	5.3	(0.21)	4.4	(0.17)	1,216	10.0	0.002	Less
CP	15.9	(0.35)	14.2	(0.28)	1,216	14.9	0.0001	Less
ST	5.8	(0.23)	5.0	(0.18)	1,216	7.2	0.008	Less
NS	7.6	(0.38)	9.4	(0.30)	1,216	13.2	0.0003	More
*Mood*								
ST	5.5	(0.15)	4.0	(0.51)	1,216	7.6	0.006	Less
Peer Rej	1.0	(0.09)	1.9	(0.31)	1,237	7.5	0.007	More
*Anxiety*								
HA	7.5	(0.31)	10.8	(0.74)	1,216	16.0	0.00009	More
ST	5.2	(0.15)	6.2	(0.36)	1,216	6.1	0.014	More
Soc Imm	1.7	(0.12)	2.8	(0.30)	1,235	11.0	0.001	More
Soc Prob	2.7	(0.19)	4.4	(0.48)	1,233	10.4	0.001	More
**Cognition** (*P* threshold. 015: handedness no effects)								
*DRD4*								
Info	12.3	(0.24)	10.9	(0.35)	1,262	11.4	0.001	Worse
SSF-max	4.9	(0.08)	5.4	(0.12)	1,255	9.7	0.002	Better
*Sex*								
CPT D-prime	0.39	(0.03)	0.26	(0.02)	1,118	15.7	0.0001	Worse
Coding	10.5	(0.31)	9.0	(0.25)	1,261	14.3	0.0002	Worse
*Mood*								
CPT Commiss	17.6	(0.90)	21.6	(0.70)	1,118	12.1	0.001	Worse
*Anxiety*								
CPT Commiss	19.5	(0.61)	23.1	(1.3)	1,118	6.2	0.014	Worse
CPT D-prime	0.33	(0.02)	0.22	(0.04)	1,118	6.7	0.011	Worse
Info	12.1	(0.21)	10.7	(0.50)	1,262	6.2	0.013	Worse

*Univariate analysis of variance was used to assess the impact of ADHD RFs on ADHD symptoms, temperament, and cognition. Each analysis reflects the effects of a specific RF after adjusting (i.e., co-varying) for the effects of age and the remaining additional RFs. See dependent measures list (Table 2) for description of measures*.

## Results

### Sample characteristics

Univariate ANOVA (SPSS v21) was used to examine each ADHD RF with respect to: ADHD symptoms, temperament, and cognitive abilities. For these analyses, a clinical, temperament, or cognitive measure of interest was entered as the dependent variable, an ADHD RF of interest was entered as the grouping variable, with age plus the remaining ADHD RFs entered as covariates in order to assess the unique impact of the ADHD RF being tested.

#### ADHD Symptoms

DRD4 7R carriers exhibited fewer inattentive symptoms. Male, mood, and anxiety RFs were associated with greater hyperactivity. Non-right handedness had no effect on symptoms (Tables 3 and Summary Table 4).

**Table 4:** **Summary of ADHD risk factor effects on clinical and cognitive measures**.

Assessments	DRDR 7R	Males	Mood affected	Anxiety affected	Non-right handed
Symptoms	Less IA	More H	More H	More H	None
Temperament	None	More NS	More Peer Rej	More HA	None
		Less RD	Less ST	More ST	
		Less CP		More Soc Imm	
		Less ST		More Soc Prob	
Cognition	Worse Info	Worse D-prime	Worse Commiss	Worse Info	None
	Better SSF-max	Worse coding		Worse D-prime	
				Worse Commiss	

*IA, inattention; H, hyperactivity*.

*For description of additional abbreviations, see Table 2*.

#### Temperament

DRD4 7R carriers and non-right handers showed no effects. Being male was associated with a less cooperative and more novelty seeking characteristics. Mood and anxiety sufferers exhibited social functioning liabilities. Anxiety also showed a strong association with harm avoidance (Table 3 and Summary Table 4).

#### Cognitive Abilities

Non-right-handers showed no effects. DRD4-7R carriers showed an enhanced ability for SWM, and worse “general knowledge,” as indexed by the WISC III information task. Males, anxiety, and mood were associated with worse CPT performance. Males showed poor CPT stimulus discrimination. Mood sufferers show poorer CPT stop inhibition. Anxiety sufferers show both poorer stimulus discrimination and stop inhibition, as well as poor “general knowledge” (Table 3 and Summary Table 4).

### Part 1: Primary analyses of right hemisphere bias in ADHD RFs

This section examines whether RH-biased brain function is a general feature of ADHF RFs. Three analyses are performed: (1) examination of parietal EEG asymmetry, (2) examination of RPA association to ADHD symptoms, and (3) examination of dichotic listening behavioral performance.

#### Step 1: Parietal EEG Asymmetry

We tested whether individual ADHD RFs were associated with increased rightward parietal EEG asymmetry (RPA) using multilevel models (software: R 2.15.2). The first level was the EEG asymmetry measure (i.e., condition and frequency). The second level was the parietal area. The third level was the family. To limit the number of tests we first examined whether there were overall effects of parietal EEG asymmetry (collapsed across specific parietal locations), then significant effects were further decomposed by looking at pairwise comparisons of individual parietal asymmetry indices.

ADHD RFs were shown to impact overall parietal EEG asymmetry. Effects were broadly expressed for DRD4 7R carriers and males, but specific for mood, anxiety, and non-right handedness (Table 5).

**Table 5:** **Overall parietal EEG asymmetry by ADHD risk factor type**.

Parietal Asym. by risk factor	Statistics		
	df	Chi-square	*P*-value
**DRD4 7R**
EC A1	3	36.6	0.00000005
EC A2	3	26.5	0.0000007
EC B1	3	10.34	0.016
EC B2	3	22.6	0.00005
EO A1	3	15.62	0.0013
EO A2	3	11.24	0.010
EO B1	3	10.94	0.012
EO B2	3	11.05	0.011
CPT A1	3	9.40	0.024
CPT A2	3	9.53	0.023
CPT B1	3	7.81	0.050
**Sex**
EC A2	3	17.77	0.0005
EC B1	3	13.54	0.003
EC B2	3	9.69	0.021
EO B1	3	18.52	0.0003
CPT A2	3	16.0	0.001
CPT B1	3	16.21	0.001
**Mood**
EC B2	3	14.76	0.002
**Anxiety**
EO B1	3	10.94	0.012
CPT B1	3	9.44	0.024
**Handedness**
EC A2	3	12.51	0.005

*Linear multilevel models and tests were to examine the overall effect of RPA by frequency and condition collapsed across three parietal brain regions. The first level was the EEG asymmetry measure (i.e., condition and frequency), the second level was the parietal area (tp8-tp7, p8-p7, p4-p3), and the third level was the family. Then for each RF, we conducted multivariate tests to examine whether it had an overall effect across the three parietal brain regions in the model. See dependent measures list (Table 2) for description of measures*.

Assessment of location-specific asymmetry indices showed that 38 of 39 significant findings exhibited RPA. RPA was broadly expressed in DRD4 7R carriers and males, but specifically expressed in mood and anxiety groups. RPA appeared strongest at the P8-7 LI. Although both DRD4 7R carriers and males showed broad RPA expression, the DRD4 7R group showed asymmetry effects mainly in alpha at the TP8-7 and P8-7 indices, while males showed asymmetry effects mainly in beta (especially beta1) across all three parietal locations. Mood only showed RPA effects during EC in beta2, while anxiety only showed RPA during active visual processing (EO, CPT) in beta1. Individuals who were not strongly right-handed did not show RPA (Table 6 and Summary Table 9).

**Table 6:** **Specific parietal EEG asymmetry indices by ADHD risk factor type**.

Asymmetry indices	No RF		With RF		Statistics			With RF asym. eff.
	$\bar{x}$	se	$\bar{x}$	se	df	*t*	*P*-value	
**DRD4 7R**								Carriers
EC A1 TP8-7	65	(20)	160	(24)	432	3.51	0.0005	R
EC A1 P8-7	70	(21)	232	(26)	432	5.68	0.00000002	R
EC A1 P4-3	50	(18)	96	(21)	432	1.97	0.049	R
EC A2 TP8-7	83	(21)	159	(25)	432	2.76	0.006	R
EC A2 P8-7	94	(22)	230	(27)	432	4.60	0.000006	R
EC B1 P8-7	57	(15)	106	(18)	431	2.47	0.014	R
EC B2 TP8-7	27	(12)	67	(14)	430	2.55	0.011	R
EC B2 P8-7	18	(13)	94	(16)	430	4.26	0.00002	R

EO A1 P8-7	67	(20)	127	(25)	395	2.15	0.032	R
EO A2 TP8-7	51	(21)	109	(26)	395	2.02	0.044	R
EO B1 TP8-7	21	(13)	67	(16)	395	2.58	0.010	R
EO B1 P8-7	33	(14)	70	(17)	395	1.95	0.050	R
EO B2 P8-7	−2	(13)	42	(16)	395	2.46	0.014	R

CPT A1 TP8-7	40	(16)	89	(21)	429	2.10	0.036	R
CPT A1 P8-7	109	(18)	179	(23)	429	2.8	0.005	R
CPT A2 TP8-7	82	(19)	139	(24)	429	2.11	0.035	R
CPT A2 P8-7	152	(21)	234	(27)	429	2.76	0.006	R
CPT B1 TP8-7	0.96	(15)	52	(19)	429	2.50	0.013	R
**Sex**								Males
EC A2 TP8-7	9	(23)	83	(21)	432	2.90	0.004	R
EC A2 P8-7	−15	(25)	94	(22)	432	3.92	0.0001	R
EC B1 TP8-7	−5	(16)	38	(15)	431	2.34	0.02	R
EC B1 P8-7	−8	(17)	57	(15)	431	3.45	0.0006	R
EC B1 P4-3	7	(12)	42	(11)	431	2.52	0.012	R
EC B2 TP8-7	−5	(14)	27	(12)	430	2.08	0.038	R
EC B2 P8-7	−27	(15)	18	(13)	430	2.65	0.008	R

EO B1 TP8-7	−34	(16)	21	(13)	395	3.23	0.0013	R
EO B1 P8-7	−25	(17)	33	(14)	395	3.18	0.0016	R
EO B1 P4-3	5	(9)	37	(8)	395	3.17	0.0016	R

CPT A2 TP8-7	11	(22)	82	(19)	429	3.23	0.004	R
CPT A2 P8-7	55	(24)	152	(21)	429	3.55	0.0004	R
CPT A2 P4-3	59	(15)	99	(14)	429	2.31	0.020	R
CPT B1 P8-7	9	(16)	72	(14)	429	3.50	0.0005	R
CPT B1 P4-3	18	(9)	48	(8)	429	3.12	0.002	R
**Mood**								Affected
EC B2 P8-7	18	(13)	112	(27)	430	3.39	0.0007	R
EC B2 P4-3	16	(8)	63	(17)	430	2.75	0.0061	R
**Anxiety**								Affected
EO B1 TP8-7	21	(13)	67	(23)	395	2.04	0.042	R
EO B1 P8-7	33	(14)	97	(25)	395	2.63	0.009	R
CPT B1 TP8-7	0.96	(15)	58	(27)	429	2.24	0.026	R
**Handedness**								Weak right- handed
EC A2 TP8-7	83	(21)	−0.99	(41)	432	2.06	0.040	L

*Pairwise comparisons of individual parietal asymmetry indices for each ADHD RF, where the above first pass test showed a general effect on parietal asymmetry (see Table 8). Like in the first pass analysis, these pairwise comparisons used linear multilevel models. The first level was the EEG asymmetry measure (i.e., condition and frequency), the second level was the parietal area (tp8-tp7, p8-p7, p4-p3), and the third level was the family*.

*Note: x-bar reflects the model adjusted means. See dependent measures list (Table 2) for description of measures*.

#### Step 2: Parietal EEG Asymmetry Association to Symptoms in ADHD RFs

Linear regression (SPSS v21) was used to examine whether RF groups interacted with individual parietal EEG asymmetry indices to predict ADHD symptoms, while controlling for age and other RF groups. Here, a symptom metric was entered as the outcome variable, then age and the “not to be examined” RF groups were entered as predictors, followed by the RF group being examined, the parietal asymmetry index being examined, and their interaction term.

Mood had no effects. All other RFs showed associations between parietal EEG asymmetry and ADHD symptoms, with anxiety showing the strongest and most numerous effects with unique weighting toward the alpha band. The findings generally demonstrated that RPA was associated with increased symptoms, except for two instances. DRD4-7R carriers showed a link between RPA and fewer inattentive symptoms, and two of eighteen significant effects for anxiety showed a link between RPA and reduced hyperactivity. In males and DRD4-7R carriers, RPA was associated with more hyperactivity. In non-right-handers, it was associated with more inattention. In anxiety, it was associated with more inattention and hyperactivity. In DRD4 7R carriers, males, and non-right handed individuals, a relationship between RPA and ADHD symptoms was mainly present for the P4-3 LI (exclusively so in males) (Table 7 and Summary Table 9).

**Table 7:** **Parietal EEG asymmetry × risk factors predicting ADHD symptoms**.

RFs × EEG asymmetry predicts	df	SB	*t*	*P*-value	With RF rightward predicts	Continued …	df	SB	*t*	*P*-value	With RF rightward predicts
**DRD4 7R**						**Anxiety continued**
*KSAD Inattention*						EC A2 P8-7	1,205	0.17	2.1	0.037	More
EC A1 P4-3	1,214	−0.20	−2.3	0.027	Less	EC B1 TP8-7	1,210	0.16	2.1	0.040	More
EO A1 P4-3	1,200	−0.20	−2.3	0.024	Less	EO A2 P8-7	1,191	0.19	2.3	0.022	More
EO B2 P4-3	1,200	−0.17	−2.0	0.05	Less	EO A2 P4-3	1,198	0.16	2.1	0.040	More
CPT B1 TP8-7	1,195	−0.18	−2.0	0.05	Less	CPT A1 P8-7	1,205	0.17	2.0	0.045	More
*KSAD Hyperactive*						CPT A2 TP8-7	1,211	0.15	2.0	0.043	More
EC B1 TP8-7	1,215	0.19	2.3	0.023	More	CPT A2 P8-7	1,205	0.18	2.2	0.030	More
CPT A2 P4-3	1,219	0.22	2.3	0.021	More	CPT A2 P4-3	1,214	0.21	2.6	0.009	More
**Male**						CPT B1 P8-7	1,205	0.21	2.6	0.010	More
*KSAD Hyperactive*						CPT B2 TP8-7	1,211	0.17	2.2	0.026	More
EC B2 P4-3	1,214	0.20	2.0	0.050	More	CPT B2 P8-7	1,205	0.28	3.9	0.0001	More
*SNAP Hyperactive*						CPT B2 P4-3	1,214	0.16	2.0	0.047	More
EC B2 P4-3	1,209	0.28	2.9	0.004	More	*KSAD Hyperactive*					
CPT A1 P4-3	1,214	0.30	2.7	0.006	More	EO A1 P4-3	1,200	−0.18	−2.3	0.021	Less
**Mood**						EO A2 P4-3	1,91	−0.34	−3.0	0.003	Less
*Nothing*						CPT B1 TP8-7	1,215	0.21	2.80	0.006	More
**Anxiety**						**Handedness**
*KSAD Inattentive*						*SNAP Inattention*					
CPT B2 TP8-7	1,215	0.20	2.8	0.006	More	EC A1 TP8-7	1,210	−0.74	−2.5	0.014	More
*SNAP Inattentive*						CPT A2 P4-3	1,214	−0.49	−2.1	0.036	More
EC A1 TP8-7	1,210	0.20	2.4	0.016	More	CPT B1 P4-3	1,214	−0.52	−2.3	0.025	More
EC A1 P8-7	1,205	0.21	2.5	0.013	More	CPT B2 P4-3	1,214	−0.45	−2.5	0.012	More

*Linear regression analysis was used to examine the interaction effects of RFs and parietal EEG asymmetry on ADHD symptoms. Each test was adjusted for the effects of age and additional RFs. See dependent measures list (Table 2) for description of measures*.

#### Step 3: Dichotic Listening Assessment of RH Bias in ADHD RFs

As a secondary behavioral validation of RH-biased processing among ADHD RFs, we examined whether ADHD RFs exhibited RH-biased signal detection during the dichotic listening paradigm, using the same univariate ANOVA procedure described in Part 1.

Mood and non-right handedness showed no effects. All significant effects showed faster responses for RF groups. DRD4 7R carriers were faster for word processing in both ears, but the effect was stronger for left-ear (RH) trials. Moreover, they did not show the standard right-ear (LH) speed advantage for word stimuli, and instead showed a left-ear (RH) advantage. DRD4-7R carriers also showed a speed advantage for detecting right-ear emotion stimuli. This trial type is understood to depend on left-to-right hemisphere transfer of emotion stimuli due to strong RH specialization for these stimuli (111). Males showed a direct left-ear (RH) speed advantage for emotion stimuli, while anxiety sufferers showed the same right-ear speed advantage for emotion stimuli as the DRD4 7R carriers (Table 8 and Summary Table 9).

**Table 8:** **Dichotic listening by ADHD risk factor type**.

Group and BD measures	No RF		With RF		Statistics			With RF
	$\bar{x}$	se	$\bar{x}$	se	df	*f*	*P*	
**DRD4 7R**
LE-word RT	1065	(19.3)	933	(27.8)	1,167	15.0	0.0001	Faster
RE-word RT	1025	(17.8)	937	(25.6)	1,167	7.8	0.006	Faster
RE-emot RT	1150	(22.9)	1068	(33.0)	1,167	4.0	0.046	Faster
**Sex**
LE-emot RT	1136	(22.8)	1072	(20.3)	1,167	4.3	0.040	Faster
**Anxiety**
RE-emot RT	1142	(20.4)	1023	(48.6)	1,167	5.0	0.027	Faster

*Univariate analysis of variance was used to assess the impact of ADHD RFs on eight dichotic listening measures (left and right performance for word and emotion targets – in accuracy and latency). All significant effects were for latency measures. Each analysis was adjusted for the effects of age and additional RFs. See dependent measures list (Table 2) for description of cognitive measures. Mood and non-right handedness showed no effects*.

**Table 9:** **Summary of right hemisphere bias assessment in ADHD RFs**.

Laterality	DRDR 7R	Males	Mood	Anxiety	Non right-handed
Parietal EEG asymmetry	All rightward	All rightward	All rightward	All rightward	1 Leftward
	18 Effects	15 Effects	2 Effects	3 Effects	EC A2 TP8-7
	Mixed cond.	Mixed cond.	Only EC	Only EO, CPT	
	Mostly alpha	Mostly beta1	Only beta2	Only beta1	
	Mainly TP8-7, P8-7	Mixed location	P4-3 and P8-7	TP8-7, P8-7	

RPA association to symptoms	Less IA. (4)	More H (3)	None	More IA (15)	More IA (4)
	More H (2)			More H (1)	
				Less H (2)	

Dichotic listening validation	RH bias indicated	RH bias indicated	None	RH bias indicated	None
	Faster word RT	Faster LE-emot		Faster RE-emot (L-to-R trans. cond.)	
	No RE-word RT adv.	
	Faster RE-emot (L-to-R trans. cond.)	

*For “RPA association to symptoms”, RPA (i.e., greater rightward parietal asymmetry) is associated with the reported outcomes. cond, condition; H, hyperactive; IA, inattentive; effs, effects; RE, right ear; LE, left ear; RT, reaction time; trans., transfer; adv., advantage; emot., emotion; “L-to-R trans. cond.” refers to the expectation that emotion stimuli presented in the right ear during this dichotic listening task are understood to undergo callosal transfer from the left to the right hemisphere, which is dominant for processing the emotion stimuli (see task description for details). For description of additional abbreviations, see Table 2*.

### Part 1: Discussion

We previously hypothesized that non-task-specialized sensory information processing should be indexed by increased weighting of RH-biased attention and visuo-perceptual brain functions during task operations, and demonstrated this phenotype in ADHD across multiple studies, using multiple methodologies [for review, see Ref. (1)]. However, as detailed in the DEP model (1), we surmised that this phenotype is not ADHD specific, but more broadly reflective of any circumstance that disrupts the induction and maintenance of an emergent task-directed neural architecture (i.e., a task-directed adaptive processing state or TD-APS). Under this view, abnormal increased weighting of RH-biased attention and visuo-perceptual brain functions is expected to generally index neurocognitive sets that are not optimized for task-directed thought and action, and when durable expressed, liability for ADHD.

The current study tested this view by examining whether previously identified rightward parietal EEG asymmetry in ADHD (22, 25, 26, 27) is generally associated with common ADHD characteristics and comorbidities (i.e., ADHD RFs). Barring one exception (non-right handedness), we found that it was. RPA was associated with carrying the DRD4-7R risk allele, being male, having mood disorder, and having anxiety disorder. This pattern was additionally supported by behavioral laterality outcomes. However, differences in the specific expression of RPA and behavioral laterality were observed, which are discussed in Part 2 in relation to possible unique mechanisms underlying ADHD liability in different ADHD RFs.

We view the single outlying handedness result with caution. Due to sample constraints, we did not test left-handedness versus right handedness, but rather the effect of being “strongly” versus “not strongly” right-handed, with the latter group including weakly right-handed, ambidextrous, and left-handed individuals. Moreover, secondary analyses implicated RH-biased processing in the “not strongly right-handed” group. Hence, we think it is reasonable to refrain from interpreting these conflicting outcomes until a better test of pure left-handedness is performed.

The otherwise clear pattern of RPA across the remaining ADHD RFs demonstrates, as predicted by the DEP model, a connection between RH-biased parietal brain function and liability for ADHD, and by inference, a general reduced capacity for task-directed thought and action. This outcome is well aligned with the established role of right-lateralized parietal brain function in attention and visual sensory information processing.

Although much remains to be elucidated about parietal brain function, multiple lines of research indicate that it plays a key role in processing information in a spatial context (113), with a dorsal-to-ventral distribution of functions related to “vision for action” versus “vision for perception” (114), and a left-to-right distribution of functions related to self-directed motoric and verbal functions (LH), versus the allocation of attention to external sensory content (RH) [for review, see Ref. (115)]. This left-right dimensionality is evident across the superior, inferior, and temporal-parietal regions examined in the current study. For instance, the LH SPL shows specialization for self-initiated fine motor actions such as writing (114, 116), whereas the RH SPL has been associated with spatial orienting (114). Similarly, the LH inferior parietal lobe (IPL) shows specialization for self-initiated grasping of objects (117), while the RH IPL is associated with orienting and sustaining attention toward external stimuli [for review, see Ref. (115, 118)].

Continuing this pattern, the LH temporal-parietal junction (TPJ) shows specialization for verbal articulatory coding (i.e., naming) (85), while the RH TPJ has been associated with stimulus-driven attentional shifting (119, 120). Related to this, Geng and Vossel (119) have recently argued that the RH TPJ plays a key role in maintaining and updating the neural context by which the relevance of incoming sensory information is vetted. This model implies that greater RH TPJ activation indexes a more flexible attentional and cognitive set (i.e., with more active updating and shifting), while reduced activation indexes a more fixed attentional and cognitive set (i.e., with less active updating and shifting). They also specify that this RH TPJ function likely draws on multiple distributed brain systems and integrates both bottom–up and top–down processing. According to this view, moment-to-moment variability in RH TPJ activation may generally index the relative degree to which individuals are oriented toward a more fixed versus flexible cognitive and attentional set.

We have previously demonstrated that ADHD is associated with abnormal increased rightward asymmetry across each of these parietal regions (P4-P3:SPL, P8-P7:IPL, TP8-TP7:TPJ) (22–25), and importantly, these outcomes could not be attributed to gender, handedness, anxiety, and/or mood. This demonstrates that ADHD involves abnormal increased weighting of right-lateralized parietal brain functions associated with: orienting attention in space (RH SPL), sustaining attention on external sensory content (RH IPL), and possibly having a more flexible attention and cognitive set (RH TPJ). Broadly speaking, these findings demonstrate an increased weighting of external-perceptual versus verbal-motoric parietal brain function in ADHD. They also highlight two possible orthogonal mechanisms that might underlie this characteristic. Greater R > L TPJ activation might index an overly flexible cognitive and perceptual set (a brain state-setting effect), while R > L SPL and IPL activations might be associated with greater attentional effort (possibly tied to compensatory effort and/or the processing of task-extraneous content).

The current study has now demonstrated that this same pattern of R > L parietal brain functions is an independent feature of multiple ADHD RFs. This ads strong support to the view that RPA may generally index: (1) brain function that is not optimized for task-directed thought and action and (2) when durably expressed, liability for ADHD.

### Part 2: Secondary analyses of RPA association to temperament and cognition in ADHD RFs

This secondary/exploratory analysis examined whether ADHD RFs exhibited different patterns of association between RPA and temperament and cognition metrics. It used the same linear regression approach described above, but with temperament and cognition metrics (rather than symptom measures) entered as outcome variables. This analysis helped us to explore whether different mechanisms (i.e., divergent/distributed deficit effects) might underlie RH-biased brain function across different ADHD RFs. General results patterns and summary tables are provide here, with full results details available in the study supplement (Tables S1 and S2 in Supplementary Material).

#### Parietal Asymmetry Association to Temperament in Different ADHD RFs

RPA showed associations to temperament characteristics in each ADHD RF. Except for males, most RPA associations to temperament occurred in the alpha-frequency band. In males, these effects were mainly in beta. Males, mood, and anxiety showed strong associations between RPA and social functioning. RPA was linked to better social functioning in males, but worse social functioning in mood and anxiety. This effect was especially pronounced for mood sufferers (43 total effects). Two effects in DRD4-7R carriers and 12 in non-right handed individuals indicated RPA association to self-oriented, rather than socially oriented, characteristics. For DRD4-7R carriers greater RPA was linked to increased self-determination and reduced novelty seeking (positive outcomes). For non-right handed individuals, greater RPA was linked to increased novelty seeking and reduced persistence and self-determination (negative outcomes). The locations of temperament effects were generally mixed, except for males (all P8-7), and non-right-hander (mainly at the TP8-7). Finally, of 31 findings involving the CPT, all but three were linked to social functioning, suggesting that this task taps brain-function characteristics important for social operations (Summary Table 10). For result details on the association between RPA and temperament characteristics, see supplement Table S1 in Supplementary Material.

**Table 10:** **Summary of RPA association with temperament by ADHD risk factor type**.

Assessments	DRD4 7R	Males	Mood	Anxiety	Non right-handed
RPA association to temperament	**Freq/Loc** (2 eff)	**Freq/Loc** (7 eff)	**Freq/Loc** (43 eff)	**Freq/Loc** (6 eff)	**Freq/Loc** (12 eff)
	All Alpha	Beta (6)	Alpha (29)	Alpha (4)	Alpha (9)
	P8-7 (1)	P8-7 (7)	TP8-7 (10)	TP8-7 (1)	TP8-7 (8)
	P4-3 (1)	**Findings**	P8-7 (21)	P8-7 (3)	P8-7 (3)
	**Findings**	Less RD (1)	P4-3 (12)	P4-3 (2)	P4-3 (1)
	Less NS (1)	Less CP (1)	**Findings**	**Findings**	**Findings**
	More SD (1)	More SD (1)	More CP (2)	Less RD (1)	More NS (2)
		Better social (4)	More ST (2)	Less SD (1)	Less PS (6)
			Worse social (39)	More HA (1)	Less SD (4)
				Worse social (3)	

*For “RPA association to temperament “more RPA (i.e., greater rightward parietal asymmetry) is associated with the reported outcomes; values in parentheses indicate the number of results (i.e., comprised of different frequency bands and/or parietal locations) showing a given effect. See Table 2 for description of abbreviations; Freq/Loc, frequency/location; eff, effects*.

#### Parietal Asymmetry Association to Cognition in Different ADHD RFs

RPA was associated with cognitive abilities in all RFs. Males and mood showed only positive effects (i.e., RPA = better performance). DRD4-7R, anxiety, and non-right handed RFs showed both positive and negative effects. A link between greater RPA and better SWM ability was evident for all groups (plus VWM in non-right handers and males). In DRD4-7R carriers, greater RPA was associated with better linguistic, but poorer attention ability. In males and mood sufferers, greater RPA was associated with better linguistic and attention ability. In anxiety sufferers, RPA was associated worse linguistic, WM, and EF ability. In non-right-handers, it was associated with worse WM and EF ability.

Associations between RPA and cognitive abilities were most evident during active visual processing (CPT and EO conditions). Males showed abundant effects at the P4-3 LI. Mood only showed effects at the TP8-7 LI. Other RFs showed mixed associations to parietal LIs. In DRD4-7R carriers, RPA associations to cognition were mostly in the alpha frequencies. Anxiety showed mixed frequency effects. Males, mood, and non-right-handers showed the majority of effects in beta frequencies. Lastly, CPT measured sustained attention was almost exclusively tied to RPA at the TP8-7 LI, while CPT and SWM tasks that required sustained attention and task-directed visual processing were predominantly linked to beta frequencies (Summary Table 11). For result details on the association between RPA and cognition, see Supplement Table S2 in Supplementary Material.

**Table 11:** **Summary of RPA Association with Cognition by ADHD Risk Factor Type**.

Assessments	DRD4 7R	Males	Mood	Anxiety	Non Right-handed
RPA association to cognition	**Freq/Loc** (15 eff)	**Freq/Loc** (18 eff)	**Freq/Loc** (13 eff)	**Freq/Loc** (15 eff)	**Freq/Loc** (29 eff)
	Alpha (11)	Beta (16)	All Beta	Beta (9)	Beta (24)
	TP8-7 (8)	TP8-7 (7)	All TP8	TP8-7 (6)	TP8-7 (12)
	P8-7 (3)	P8-7 (3)	**Better** (13 eff)	P8-7 (8)	P8-7 (15)
	P4-3 (4)	P4-3 (8)	St-Word (1)	P4-3 (1)	P4-3 (1)
	**Better** (8 eff)	**Better** (18 eff)	SWM Acc (5)	**Better** (3 eff)	**Better** (15 eff)
	Read_rec (2)	Read_rec (1)	SWM RTSD (1)	SSF-acc (1)	DSB-acc (1)
	St-Word (1)	DSF-acc/max (3)	Omiss (2)	SWM RTSD (2)	SWM Acc (14)
	Arith (2)	SSF-acc/max (5)	D-prime (3)	**Worse** (12 eff)	**Worse** (14 eff)
	SWM RTSD (3)	SSB-acc (1)	Bias (1)	Read_rec (2)	ST-Inter (1)
	**Worse** (7 eff)	SWM Acc (3)		Phono (1)	DSB-max (1)
	Omiss (3)	SWM RTSD (3)		Coding (1)	SWM RT (12)
	Hit RTSE (1)	Hit RTSE (1)		St-Inter (4)	
	Bias (3)	D-prime (1)		DSF-acc/max (3)	
				SSB-max (1)	

*For “RPA association to cognition” more RPA (i.e., greater rightward parietal asymmetry) is associated with the reported outcomes; Values in parentheses indicate the number of results (i.e., comprised of different frequency bands and/or parietal locations) showing a given effect. See Table 2 for description of measures abbreviations; Freq/Loc, frequency/location; eff, effects*.

### Part 2 discussion

The DEP model of ADHD highlights that multiple distributed brain systems get dynamically coordinated during task-directed thought and action. We posited that these systems comprise an emergent task-directed neural architecture (i.e., the TD-APS) that mobilizes RH resources in a specific task-specialized manner that facilitates fast (i.e., economical) identification of task-relevant content. Any impairment to this system, no matter the cause, is expected to reduce this capacity, and produce an associated increased expression of RH attention and non-task-entrained visuo-perceptual processing. Variable deficits to this system are expected to underlie ADHD heterogeneity, and manifest variant forms of this shared characteristic. The model also postulates that variable primary deficits might be elucidated by examining the relationship between RH-biased processing and constituent system operations. We explored this possibility in our secondary analyses.

As predicted, different ADHD RFs showed variant expressed forms of RPA, along with different patterns of association between RPA and clinical and cognitive metrics. This supports the view that RPA is tied to different mechanisms in different ADHD RFs. The current study design cannot resolve what these are; however, through consideration of our total study findings, we can begin to speculate about possible differences in this regard. The following discussion of this topic and associated findings is highly speculative, but is provided to help stimulate further conceptual and experimental analysis of this topic.

The most obvious difference across ADHD RFs was that DRD4-7R and males exhibited RPA in multiple brain regions, frequencies, and conditions, whereas mood and anxiety showed very specific effects. One possible explanation is that genetically linked risk for ADHD (DRD4-7R carriers and males) is tied to more generalized and stable brain-function characteristics, while more experience-linked conditions (mood and anxiety) involve more state and/or circumstance specific abnormal brain functioning. Regardless, this pattern indicates that categorically different forms of brain function may underlie RPA in more genetically versus experience weighted conditions.

Within these domains, additional unique RPA characteristics were evident. The DRD4-7R group was predominantly associated with RPA in the alpha band (11/18 effects in alpha), while being male was predominantly associated with RPA in the beta band (10/15 effects in beta). According to recent conceptualizations of these frequencies, this might indicate increased RH internal-processing control in DRD4-7R carriers (121–123), but greater expression of RH visuo-perceptual functioning in males [for review, see Ref. (124–126)].

Behavioral laterality outcomes appear to support this speculation. DRD4-7R carriers showed an atypical pattern of being faster for word targets in the right versus left hemisphere (111, 127). They also showed faster reaction times for a specific dichotic listening trial type that is understood to require left-to-right callosal transfer of information due to exclusive RH competence for the stimuli (i.e., detecting the negative tone of voice in the right-ear projecting initially to the LH). Both results are consistent with greater RH processing control in DRD4-7R carriers (111). In contrast, being male was associated with faster responses for the trial type that directly taps RH ability (i.e., detecting the negative tone of voice in the left-ear projecting directly to the RH). In sum, both RPA and behavioral laterality findings seem consistent with greater RH processing control in DRD4-7R carriers, but greater expression of RH visuo-perceptual functions in males.

Mood and anxiety also differed in their expression of RPA and behavioral laterality outcomes. In mood, RPA was only evident during the EC condition (with no active sensory engagement). In anxiety, it was only evident during the EO and CPT conditions (with active sensory engagement). Consistent with this, mood showed no behavioral laterality effects during the dichotic listening task that requires active sensory processing, while anxiety was associated with faster responses for the trial type (noted above) that requires left-to-right transfer of information (111). Hence, RPA and behavioral laterality outcomes suggest that R > L parietal brain function may only be evident in mood sufferers when they are not sensory-engaged, and in anxiety sufferers when they are. This pattern, along with the above described variant expressions of RPA in DRD4-7R carriers and males, generally supports the view that unique mechanisms may underlie poor task-directed brain function and RPA across different ADHD RFs.

#### Additional Specific Hypotheses

In order to generate more specific hypothesis about the nature of abnormal brain function across ADHD RFs, we will now consider individual RFs clinical characteristics, and patterns of RPA association to symptoms, temperament, and cognitive abilities. This analysis is again speculative, but is presented to help generate testable hypotheses for future analysis.

##### The DRD4-7R allele

Carrying the DRD4-7R allele was generally distinguished by having more positive outcomes, and RPA effects being mainly in the alpha band. It was associated with having fewer inattentive symptoms and an advantage for spatial working memory. RPA (mainly in alpha) was associated with fewer inattentive symptoms, positive temperament characteristics, and better ability for tasks amenable to visual strategies. Negative RPA associations were exclusive to the CPT, and indicated a link to impulsive and variable performance.

The CPT requires verbal encoding and maintaining a task-directed set for a prolonged period (i.e., 14 min). This perhaps makes the CPT less amenable to alternative RH strategies. Moreover, negative RPA-CPT performance associations only occurred for RPA in the TPJ (i.e., TP8-TP7). This may indicate a link between cognitive flexibility and poor CPT performance in this ADHD RF. Given this pattern, and indications of strong positive selection for the DRD4-7R allele (128), we speculate that this RF might reflect an evolved specialization for more flexible visual cognition that supports exploratory behavior, but is deleterious for task-directed actions.

##### Being male

Being male was generally distinguished by poorer verbal ability, and by RPA effects occurring mainly in the beta band. Males exhibited worse letter discrimination and coding ability. RPA (mainly in beta) was associated with greater hyperactivity, better social functioning, and better cognitive ability (e.g., spatial, verbal, and less response variability). As with the DRD4-7R carriers, we speculate that being male is linked to an evolved emphasis on RH-biased brain function that is less conducive to task-directed thought and behavior, albeit in a different manner.

EEG beta has been linked to early-stage encoding of attentionally selected information [for review, see Ref. (124–126)] and shows lateralized posterior expression with verbal and visual-spatial processing (129, 130). Hence, the emphasis of EEG beta and poorer verbal ability in males (74, 75) seems consistent with greater weighting of RH-biased sensory encoding. In this vein, greater RH visual-cortical volumes have been identified in males (131), and abundant gender differences in visual processing are identified [for review, see Ref. (132, 133)].

Women exhibit greater specialization for top–down object-oriented processing (i.e., categorization/naming), while men emphasize bottom–up analysis of motion, fine detail, and high resolution spatial and temporal content [for review, see Ref. (132, 134)]. Moreover, a recent study has demonstrated that while the female style of sensory processing is well adapted to EF-demanding dual-task paradigms, the male style is not (132). Given this literature and our current findings, we speculate that risk for ADHD in males is linked to greater expression of RH bottom–up visual processing, which is generally less conducive to the induction and/or maintenance of a task-directed neural architecture. Perhaps consistent with this male RPA effects on cognition were wholly positive, including for CPT metrics, suggesting that compensatory attentional effort might be an important driver of RPA in males.

##### Having mood disorder

Having mood disorder was distinguished by greater inattention, poorer inhibition, RPA being exclusively expressed during the EC condition, and no RPA associations to symptoms, but abundant RPA associations to poor social functioning. For example, mood sufferers exhibited greater CPT commission errors (i.e., poor response inhibition). Poor inhibition is consistent with RH frontal pathology in mood disorders (57, 135). Moreover, mood was the only RF that did not exhibit RPA association to symptoms; however, they exhibited far more RPA associations to bad social outcomes than any other group. Finally, as in males, RPA associations to cognition were exclusively positive.

Taken together, we speculate that RH bias in mood sufferers, identified here and elsewhere [for review, see Ref. (135)], may reflect internal affective processing (i.e., rumination) that imparts reduced awareness of external cues needed to guide attention in a socially adaptive manner (i.e., poor entrainment of attention functions to external social contexts). Perhaps consistent with this, mood-linked RPA was only evident in SPL and IPL regions, which are often linked to applied attentional effects.

Furthermore, greater R > L TPJ activation was tied to better cognitive outcomes for the mood RF. It is interesting to consider that a more flexible cognitive set might help mood sufferers be more responsive to external social cues, and thereby make socially appropriate transitions into task-directed cognitive sets. If true, mood derived risk for ADHD should be moderated by factors that promote more flexible and/or externally oriented cognitive and attentional sets (e.g., fluid intelligence, creative ability, self-transcendence). Consistent with this, in our ADHD sample, comorbid mood was associated with low self-transcendence (i.e., feeling less connected to the external environment) (136).

##### Having anxiety disorder

Having anxiety disorder was distinguished by being the most impaired RF, by RPA occurring only during sensory engagement, and by having the most numerous RPA associations to symptoms and poor cognition. The DRD4-7R group was associated with reduced general knowledge (information task), being male was associated with poorer verbal ability, and mood was associated with poor inhibition, having anxiety disorder was associated with each. It was also tied to greater hyperactivity, harm avoidance, and social problems. RPA showed the most numerous associations with symptoms, marginal association to poor social functioning, and mixed effects on cognition, but with the most numerous negative effects. Different from DRD4-7R carriers, anxiety sufferers showed a standard pattern of RPA association to positive outcomes for spatial tasks, and poor outcomes for standard verbal tasks and the CPT. Hence, rather than increased RH processing control, we speculate that anxiety imparts a more fight-or-flight like increase of bottom–up visuo-perceptual processing. If true, anxiety may produce a similar, albeit more severe, version of the ADHD liability that we suggest occurs in males (i.e., greater expression of bottom–up visual processing).

In considering the sum total of our findings, along with relevant literature, we speculate that RPA is tied to different causal mechanisms in different ADHD RFs. We suspect that DRD4-7R carriers may adhere to visual cognition strategies that bear liability for tasks that are not amenable to visual cognition strategies. Males and anxiety sufferers may bear an increased tendency toward bottom–up visual processing (automatic sensory responsivity) that makes the induction and maintenance of a task-directed neural architecture more difficult (especially for anxiety sufferers). We suspect that in males this is typically overcome, but nevertheless imparts risk for ADHD, while in anxiety sufferers, it more consistently underlies attention related pathology. Finally, we speculated that internal affective processing in mood sufferers might diminish entrainment to social cues needed to guide attention.

## General Summary and Conclusion

The DEP model conceptualizes ADHD to be a set of clinical characteristics that manifest with any form of poor task-directed brain functioning (i.e., convergent effects). It asserts that R > L posterior brain function is a similarly manifest physiologic outcome. We tested this by examining whether R > L parietal EEG asymmetry was generally associated with common ADHD RFs, and found that it was. This suggests that ADHD liability is generally associated with increased weighting of RH visuo-perceptual versus LH verbal parietal brain functions, and highlights two possible mechanisms: (1) a TPJ state-setting effect and (2) an SPL/IPL applied attention effect. Moreover, we hypothesize that abnormal RH contribution in ADHD during task operations can be conceived to include: (1) reduced top–down task-directed control over visual processing (low RH), (2) a more diffuse/flexible attentional and cognitive set, associated with greater expression of non-task-specialized forms of visual processing (high RH), and (3) compensatory attentional effort (high RH).

In secondary analyses, we explored whether RPA (a convergent feature of ADHD liability) might be tied to different causal mechanisms in different ADHD RFs. Different patterns of RPA and RPA association to symptoms, temperament, and cognition suggest that it is. We suspect that ADHD RFs disrupt the induction and/or maintenance of a task-optimized neural architecture (i.e., a TD-APS), albeit in different manners. We also expect that multiple additional acute (e.g., sleep deprivation, highly engaging sensory experience, emergencies, etc.), developmental (e.g., childhood trauma/abuse), genetic (e.g., that promote a novelty/risk-taking temperament), and/or mechanistic (e.g., brain injury, learning disabilities) factors can produce this outcome. Under this view, ADHD symptoms are considered to reflect the convergent or shared outcome of poor task-directed brain functioning, no matter the cause, with the syndrome of ADHD reflecting such circumstances that get durably expressed and that are life disruptive.

Viewed in this way, it is clear that the challenge of understanding and treating ADHD is, at its core, synonymous with the challenge of understanding complex task-directed thought and action, and the multitude of ways that ability might be compromised. It also highlights that identification of primary deficits may require a greater emphasis on longitudinal analysis of ADHD individuals and/or clinically meaningful subgroups. However, as demonstrated by current drug treatments, alleviation of ADHD symptoms may not require a complete knowledge of deficit causality.

Given the diffuse anatomy of catecholomine systems, stimulants likely impart broad, rather than specific effects (137). One possibility is that they help to generally nudge brain function toward computational states that are conducive to task-directed thought and action, and thus provide a degree of clinical benefit regardless of diverse deficit sources. Moreover, the two primary systems targeted by ADHD drug therapies, dopamine and norepinephrine, appear to exhibit a degree of left and right hemisphere specialization (77, 138), which may also align with identified left–right dichotomies related to internal-verbal versus external-perceptual brain functions (129). It is interesting to consider that stimulants might help to achieve a task-optimized leftward balance along these continuums (i.e., greater brain-state orientation toward internal-verbal versus external-perceptual processing). If true, additional non-drug based therapies aimed at bolstering verbal and internally weighted brain function may prove helpful for ADHD individuals. To this end, we recently used transcranial magnetic stimulation (TMS) to increase relative LH parietal contribution (near TPJ) in ADHD adults, and obtained highly encouraging preliminary results. We think that cognitive training strategies previously shown to increase bias toward LH verbal-categorical processing [for review, see Ref. (139)] should also be explored.

In sum, the current study and DEP model of ADHD have attempted to highlight, and begin to address, the challenges associated with trying to map sharply defined diagnostic categories onto underlying pathophysiology, as abnormal brain-function characteristics are often shared by multiple disorders. To this end, aberrant rightward asymmetry has been reported across multiple conditions that are linked to attention difficulties, and that are often comorbid with ADHD (e.g., anxiety, depression, sleep deprivation, novelty seeking, reading disability, etc.) (8, 131, 132, 140–147). Our core assertion is that this phenotype is generally linked to brain-function dynamics that are not optimized for complex multi-step and/or contingency-based task-directed sensory processing and cognition. Accordingly, and as noted, we expect that the effects observed in the current study can occur independent of an ADHD diagnosis; however, and importantly, we do not expect them to be independent of attentional liability for complex task-directed brain-function. Whether or not an ADHD diagnosis gets applied is a matter of clinical judgment.

## Conflict of Interest Statement

The authors declare that the research was conducted in the absence of any commercial or financial relationships that could be construed as a potential conflict of interest.

## Supplementary Material

The Supplementary Material for this article can be found online at http://journal.frontiersin.org/article/10.3389/fpsyt.2015.00063
