# Supplementary material for: A Parietal Biomarker for ADHD Liability: As Predicted by the Distributed Effects Perspective Model of ADHD
Source: Front Psychiatry. 2015 May 7;6:63. doi: 10.3389/fpsyt.2015.00063 (PMC4423436; doi:10.3389/fpsyt.2015.00063)
Supplement: Supplementary file 1 [file datasheet_1.PDF]

Supplement
Part-2 Secondary Results Details And Summary of All Study Findings

Table S1. Parietal EEG Asymmetry x Risk Factors Predicting Temperament

RFs x EEG Asymmetry Predicts	df	SB	t	p-val	With RF Rightward Predicts
**DRD4 7R**					
Novelty Seeking					
**- EO A1 P8-7**	1,168	-.27	-2.7	.009	less
Self Determination					
- EO A1 P4-3	1,174	.24	2.6	.011	more
**Male**					
Reward Dependence					
- CPT B2 P8-7	1,171	-.30	-2.4	.016	less
Cooperativeness					
- CPT A1 P8-7	1,171	-.35	-2.6	.009	less
Social Immaturity					
**- EC B2 P8-7**	1,185	-.26	-2.5	.015	less
- EO B2 TP8-7	1,177	-.39	-3.0	.003	less
Peer Rejection					
**- EC B2 P8-7**	1,187	-.28	-2.7	.008	less
Social Problems					
**- EC B2 P8-7**	1,183	-.28	-2.7	.007	less
Self Determination					
- EO B2 P8-7	1,168	.31	2.7	.009	more
**Mood**					
Cooperativeness					
- EO A1 P4-3	1,174	.23	2.5	.012	more
- CPT A1 P4-3	1,181	.22	2.6	.010	more
Self Transcendence					
- EO A1 P4-3	1,174	.25	2.8	.005	more
- EO A2 P4-3	1,174	.21	2.5	.013	more
Social Immaturity					
- CPT A1 TP8-7	1,195	.20	2.9	.005	more
- CPT A1 P8-7	1,187	.23	2.9	.004	more
- CPT A1 P4-3	1,197	.21	2.6	.009	more
- CPT A2 TP8-7	1,195	.20	2.8	.006	more
- CPT A2 P8-7	1,187	.23	2.8	.005	more
- CPT A2 P4-3	1,197	.24	2.8	.005	more
- CPT B1 P8-7	1,187	.23	3.0	.003	more
- CPT B1 P4-3	1,197	.26	2.8	.005	more
- CPT B2 P4-3	1,197	.19	2.5	.014	more
Peer Rejection					
- EC A1 TP8-7	1,194	.20	2.5	.015	more
- EC A1 P8-7	1,187	.26	3.0	.003	more
- EC A2 P8-7	1,187	.26	3.0	.003	more
- EC B1 P8-7	1,187	.23	3.1	.002	more
**- EC B2 P8-7**	1,187	.23	2.7	.008	more
- EO A2 TP8-7	1,177	.19	2.7	.007	more
- EO B1 P8-7	1,175	.21	2.5	.015	more
- EO B2 P4-3	1,181	.22	2.7	.007	more
- CPT A1 TP8-7	1,195	.18	2.6	.010	more
- CPT A1 P8-7	1,188	.20	2.5	.012	more
- CPT A2 TP8-7	1,195	.23	3.3	.001	more
- CPT A2 P8-7	1,188	.29	3.6	.0004	more

Continued …	df	SB	t	p-val	With RF Rightward Predicts
**Mood Continued**					
- CPT A2 P4-3	1,198	.23	2.6	.010	more
- CPT B1 P8-7	1,188	.32	4.3	.00003	more
- CPT B2 P8-7	1,188	.19	2.6	.010	more
Social Problems					
- EC A1 TP8-7	1,190	.21	2.5	.014	more
- EC A1 P8-7	1,183	.24	2.8	.006	more
- EC A2 P8-7	1,183	.26	3.0	.003	more
- EC B1 P8-7	1,183	.21	2.8	.006	more
**- EC B2 P8-7**	1,183	.24	2.7	.007	more
- EO A2 TP8-7	1,175	.20	2.7	.006	more
- EO A2 P8-7	1,173	.23	2.5	.014	more
- CPT A1 TP8-7	1,193	.22	3.2	.002	more
- CPT A1 P8-7	1,185	.26	3.2	.001	more
- CPT A2 TP8-7	1,193	.24	3.4	.001	more
- CPT A2 P8-7	1,185	.29	3.6	.0003	more
- CPT A2 P4-3	1,195	.27	3.1	.002	more
- CPT B1 P8-7	1,185	.30	3.9	.0001	more
- CPT B1 P4-3	1,195	.26	2.8	.005	more
- CPT B2 P8-7	1,185	.20	2.6	.009	more
**Anxiety**					
Reward Depend					
- EC A1 P4-3	1,179	-.22	-2.6	.011	less
Self Determination.					
- CPT B2 TP8-7	1,179	-.19	-2.5	.015	less
Harm Avoidance					
- EO A2 P4-3	1,174	.20	2.6	.010	more
Social Immaturity					
- EO B2 P8-7	1,175	.20	2.5	.014	more
Peer Rejection					
- EC A1 P8-7	1,204	.25	3.1	.002	more
Social Problems					
- EC A1 P8-7	1,183	.24	2.9	.004	more
**Handedness**					
Novelty Seeking					
- EO A2 TP8-7	1,169	-1.1	-3.1	.002	more
- CPT A1 TP8-7	1,179	-.51	-2.6	.010	more
Persistence					
- EC A1 TP8-7	1,180	.72	2.9	.004	less
- EC A1 P8-7	1,176	.77	3.3	.001	less
- EC A2 P8-7	1,176	.55	2.7	.008	less
- EC B1 TP8-7	1,180	.53	2.5	.012	less
- EC B2 TP8-7	1,179	.38	2.7	.008	less
- EO A2 TP8-7	1,169	1.1	3.1	.002	less
Self Determination					
- EC A1 P8-7	1,176	.66	2.9	.004	less
- EO A2 TP8-7	1,169	.86	2.5	.013	less
- CPT A1 TP8-7	1,179	.53	2.6	.010	less
- CPT B2 P4-3	1,181	.57	2.9	.004	less

Temperament effects reported at a p threshold of .015. Linear regression analysis was used to examine the interaction effects of RFs and parietal EEG asymmetry on temperament characteristics. Each test was adjusted for the effects of age and additional RFs. See dependent measures list (table 2) for description of measures. Bold signifies AI measures that showed significant rightward asymmetry for a given group. Italics signify AI measures that showed significant association between RPA and ADHD symptoms for a given group.

Table S2. Parietal EEG Asymmetry x Risk Factor Predicting Cognition- Part 1

RFs x EEG Asymmetry Predicts	df	SB	t	p-val	With RF Rightward Predicts
**DRD4 7R**					
Reading Recog.					
- EO A2 P8-7	1,195	.24	2.4	.015	better
- EO A2 P4-3	1,202	.23	2.6	.009	better
St-Word Naming					
- CPT A2 P4-3	1,204	.18	2.5	.013	better
Arithmetic					
- EO A2 P8-7	1,192	.25	2.5	.014	better
- EO A2 P4-3	1,199	.22	2.5	.014	better
SWM L3 RTSD					
**- EO A2 TP8-7**	1,127	-.35	-3.3	.001	better
- EO A2 P8-7	1,123	-.35	-2.8	.006	better
- EO A2 P4-3	1,130	-.35	-3.3	.001	better
CPT Omissions					
- EO A1 TP8-7	1,87	.38	3.7	.0004	worse
**- EO B1 TP8-7**	1,87	.30	2.7	.008	worse
- EO B2 TP8-7	1,87	.30	2.8	.006	worse
CPT Hit RTSE					
- EO A1 TP8-7	1,87	.34	3.2	.002	worse
CPT Bias					
- EO A1 TP8-7	1,86	-.40	-3.8	.0003	worse
**- EO B1 TP8-7**	1,86	-.30	-2.6	.012	worse
- EO B2 TP8-7	1,86	-.33	-3.0	.003	worse
**Male**					
Reading Recog.					
**- CPT B1 P4-3**	1,218	.32	2.6	.009	better
Digit Span Fr Acc					
**- EO B1 P4-3**	1,181	.33	2.6	.010	better
- EO B2 P4-3	1,181	.37	3.0	.003	better
Digit Span Fr Max					
- EO B2 P4-3	1,198	.27	2.7	.007	better
Spatial Span Fr Acc					
**- EC B2 P8-7**	1,208	.28	2.8	.006	better
**- EO B1 P4-3**	1,200	.30	2.5	.013	better
- EO B2 P4-3	1,200	.38	3.6	.0004	better
Spatial Span Fr Max					
**- EO B1 P4-3**	1,200	.26	2.6	.011	better
- EO B2 P4-3	1,200	.30	3.3	.001	better
Spatial Span Br Acc					
**- CPT A2 TP8-7**	1,211	.31	2.8	.005	better
**Continued …**					
**Male Continued**					
CPT Hit RTSE					
**- CPT A2 TP8-7**	1,104	-.38	-2.5	.015	better
CPT Sensitivity					
**- EC B2 TP8-7**	1,90	.43	2.4	.016	better
SWM Load 1 Acc					
- EO B2 P8-7	1,123	.34	2.9	.004	better
SWM Load 1 RTSD					
- CPT B2 P8-7	1,136	-.29	-2.7	.008	better
SWM Load 3 RT					
- EC A2 P4-3	1,128	.36	2.9	.004	slower
SWM Load 5 Acc					
**- EC B2 TP8-7**	1,129	.38	2.9	.004	better
**- EO B1 TP8-7**	1,127	.33	2.5	.012	better
SWM Load 5 RT					
- EC A2 P4-3	1,128	.42	3.2	.002	slower
SWM Load 7 RTSD					
**- EC B1 TP8-7**	1,129	-.37	-2.9	.004	better
- CPT B1 TP8-7	1,142	-.44	-3.2	.002	better
**Mood**					
St-Word Naming					
- EO B1 TP8-7	1,188	.15	2.8	.005	better
CPT Omissions					
- EO B1 TP8-7	1,87	-.32	-3.2	.002	better
- EO B2 TP8-7	1,87	-.33	-3.0	.004	better
CPT Sensitivity					
- EO B1 TP8-7	1,86	.34	3.5	.001	better
- EO B2 TP8-7	1,86	.35	3.2	.002	better
- CPT B1 TP8-7	1,103	.27	3.2	.002	better
CPT Bias					
- EO B1 TP8-7	1,86	.29	2.8	.006	better
SWM Load 1 RTSD					
- EC B1 TP8-7	1,129	-.21	-2.6	.010	better
SWM Load 5 Acc					
- EO B1 TP8-7	1,127	.26	3.0	.003	better
- EO B2 TP8-7	1,127	.27	3.2	.002	better
- CPT B1 TP8-7	1,142	.19	2.5	.013	better
SWM Load 7 Acc					
- EO B1 TP8-7	1,127	.22	2.6	.009	better
- EO B2 TP8-7	1,127	.24	3.0	.003	better

Cognition outcomes reported at p threshold of .015. Part-1: Linear regression analysis was used to examine the interaction effects of RFs and parietal EEG asymmetry on ADHD subject's cognitive abilities. Each test was adjusted for the effects of age and additional RFs. See dependent measures list (table 2) for description of measures. Bold signifies AI measures that showed significant rightward asymmetry for a given group. Italics signify AI measures that showed significant association between RPA and ADHD symptoms for a given group.

Table S2 Continued: Parietal EEG Asymmetry x Risk Factor Predicting Cognition- Part 2

**RFs x EEG Asymmetry Predicts**	df	SB	t	p-val	With RF Rightward Predicts
**Anxiety**					
Reading Recog.					
- EC A1 TP8-7	1,217	-.23	-2.8	.006	worse
- EC B1 P8-7	1,211	-.19	-2.6	.011	worse
Phonologic					
- EO A1 TP8-7	1,195	-.21	-2.5	.012	worse
Stroop Interference					
- EO A1 P8-7	1,176	-.26	-3.0	.004	worse
- EO A2 TP8-7	1,176	-.21	-2.5	.013	worse
- EO A2 P8-7	1,176	-.31	-3.7	.0003	worse
- CPT A1 TP8-7	1,186	-.22	-2.6	.009	worse
Digit Span Fr Acc					
- EC B2 TP8-7	1,192	-.24	-3.0	.003	worse
- EC B2 P8-7	1,187	-.27	-3.1	.002	worse
Digit Span Fr Max					
- EC B2 P8-7	1,205	-.18	-2.6	.009	worse
Coding					
- EO B2 P8-7	1,194	-.21	-2.7	.008	worse
Spatial Span Bk Max					
- EO B2 P4-3	1,200	-.20	-2.8	.005	worse
Spatial Span Fr Acc					
- CPT B1 TP8-7	1,211	.24	3.0	.003	better
SWM Load 3 RTSD					
**- EO B1 P8-7**	1,123	-.32	-3.0	.003	better
SWM Load 7 RTSD					
**- EO B1 P8-7**	1,123	-.30	-2.9	.005	better
**Handedness**					
Stroop Interfer.					
- EC A2 TP8-7	1, 189	.55	2.67	.008	worse
Digit Span Bk Max					
- EC A2 TP8-7	1,211	.39	2.49	.014	worse
SWM Load1 RT					
- EC B2 P8-7	1,124	-.41	-2.6	.011	slower
- EO B2 P8-7	1,123	-.42	-2.6	.011	slower

**Continued …**	df	SB	t	p-val	With RF Rightward Predicts
**Handedness Continued …**					
SWM Load 3 RT					
- EC B2 P8-7	1,124	-.48	-2.9	.004	slower
- EO B2 TP8-7	1,127	-.57	-2.9	.004	slower
- EO B2 P8-7	1,123	-.64	-3.7	.0003	slower
- EO B2 P4-3	1,130	-.61	-2.6	.011	slower
SWM Load 5 RT					
- EC B2 TP8-7	1,129	-.37	-2.9	.005	slower
- EC B2 P8-7	1,124	-.68	-3.8	.0002	slower
- EO B2 TP8-7	1,127	-.62	-3.0	.004	slower
- EO B2 P8-7	1,123	-.63	-3.3	.001	slower
SWM Load 7 RT					
- EC B2 P8-7	1,124	-.60	-3.5	.001	slower
- EO B2 P8-7	1,123	-.59	-3.2	.002	slower
Digit Span Bk Acc					
- EC B1 P4-3	1,191	.45	2.6	.010	better
SWM Load 1 Acc					
- EC B2 P8-7	1,124	-.49	-2.6	.009	better
- EO B2 P8-7	1,123	-.66	-3.5	.001	better
SWM Load 3 Acc					
- EC B2 P8-7	1,123	-.53	-2.9	.004	better
- EO B2 P8-7	1,123	-.57	-3.1	.002	better
SWM Load 5 Acc					
- EO A1 TP8-7	1,127	-.81	-3.1	.002	better
- EO A1 P8-7	1,123	-.76	-3.0	.003	better
- EO B1 TP8-7	1,127	-.62	-3.0	.003	better
- EO B2 TP8-7	1,127	-.59	-2.8	.007	better
- CPT B1 TP8-7	1,142	-.51	-2.9	.005	better
SWM Load 7 Acc					
- EC B2 TP8-7	1,129	-.31	-2.5	.015	better
- EC B2 P8-7	1,124	-.45	-2.5	.014	better
- EO B1 TP8-7	1,127	-.55	-2.8	.006	better
- EO B2 P8-7	1,123	-.46	-2.5	.014	better
- CPT B1 TP8-7	1,142	-.53	-3.1	.002	better

Cognition outcomes reported at p threshold of .015. Part-2: Linear regression analysis was used to examine the interaction effects of RFs and parietal EEG asymmetry on ADHD subject's cognitive abilities. Each test was adjusted for the effects of age and additional RFs. See dependent measures list (table 2) for description of measures. Bold signifies AI measures that showed significant rightward asymmetry for a given group. Italics signify AI measures that showed significant association between RPA and ADHD symptoms for a given group.

Table S3: Summary of All Results Patterns

Assessments	DRD4 7R	Males	Mood	Anxiety	Non R-handed
**General Characteristics**	- Less IA<br>- No temperament effs<br>- Worse Info<br>- Better SSF-max	- More H<br>- Less RD, CP, ST<br>- More NS<br>- Worse D-prime<br>- Worse Coding	- More IA<br>- Less ST<br>- More Peer Rej<br>- Worse Commiss	- More H<br>- More: HA, ST<br>- More Soc Imm/Prob<br>- Worse Info<br>- Worse D-prime<br>- Worse Commiss	- None
**Parietal Asymmetry**	**All Rightward**<br>- 18 effs<br>- Mixed Conditions<br>- Mostly Alpha<br>- Mixed LI (only 1 P4-3)	**All Rightward**<br>- 15 effs<br>- Mixed Conditions<br>- Mostly Beta 1<br>- Mixed LI (4 at P4-3)	**All Rightward**<br>- 2 effs<br>- EC B2 P8-7<br>- EC B2 P4-3	**All Rightward**<br>- 3 effs<br>- EO B1 TP8-7<br>- EO B1 P8-7<br>- CPT B1 TP8-7	**Leftward**<br>- 1 eff<br>- EC A2 TP8-7
**RPA Association To Symptoms**	- Less IA. (4 eff)<br>- More H (2 eff)	- More H (3 eff)	- None	- More IA (14 eff)<br>- More H (2 eff)<br>- Less H (2 eff)	- More IA (4 eff)
**BD Laterality Behavioral Validation**	**RH Bias Indicated**<br>- Words reversed asym<br>- Word faster LE-RH<br>- Emot Faster L-to-R	**RH Bias Indicated**<br>- Emot faster in LE-RH	- None	**RH Bias Indicated**<br>- Emot Faster L-to-R	- None
**RPA Association To Temperament**	**Freq/Loc** (2 eff)<br>- All Alpha<br>- P8-7 (1)<br>- P4-3 (1)<br>**Findings**<br>- Less NS (1)<br>- More SD (1)	**Freq/Loc** (7 eff)<br>- Beta (6)<br>- P8-7 (7)<br>**Findings**<br>- Less RD (1)<br>- Less CP (1)<br>- More SD (1)<br>- Better Social (4)	**Freq/Loc** (43 eff)<br>- Alpha (29)<br>- TP8-7 (10)<br>- P8-7 (21)<br>- P4-3 (12)<br>**Findings**<br>- More CP (2)<br>- More ST (2)<br>- Worse Social (39)	**Freq/Loc** (6 eff)<br>- Alpha (4)<br>- TP8-7 (1)<br>- P8-7 (3)<br>- P4-3 (2)<br>**Findings**<br>- Less RD (1)<br>- Less SD (1)<br>- More HA (1)<br>- Worse Social (3)	**Freq/Loc** (12 eff)<br>- Alpha (9)<br>- TP8-7 (8)<br>- P8-7 (3)<br>- P4-3 (1)<br>**Findings**<br>- More NS (2)<br>- Less PS (6)<br>- Less SD (4)
**RPA Association To Cognition**	**Freq/Loc** (15 eff)<br>- Alpha (11)<br>- TP8-7 (8)<br>- P8-7 (3)<br>- P4-3 (4)<br>**Better** (8 eff)<br>- Read_rec (2)<br>- St-Word (1)<br>- Arith (2)<br>- SWM RTSD (3)<br>**Worse** (7 eff)<br>- Omiss (3)<br>- Hit RTSE (1)<br>- Bias (3)	**Freq/Loc** (18 eff)<br>- Beta (16)<br>- TP8-7 (7)<br>- P8-7 (3)<br>- P4-3 (8)<br>**Better** (18 eff)<br>- Read_rec (1)<br>- DSF-acc/max (3)<br>- SSF-acc/max (5)<br>- SSB-acc (1)<br>- SWM Acc (3)<br>- SWM RTSD (3)<br>- Hit RTSE (1)<br>- D-prime (1)	**Freq/Loc** (13 eff)<br>- All Beta<br>- All TP8<br>**Better** (13 eff)<br>- St-Word (1)<br>- SWM Acc (5)<br>- SWM RTSD (1)<br>- Omiss (2)<br>- D-prime (3)<br>- Bias (1)	**Freq/Loc** (15 eff)<br>- Beta (9)<br>- TP8-7 (6)<br>- P8-7 (8)<br>- P4-3 (1)<br>**Better** (3 eff)<br>- SSF-acc (1)<br>- SWM RTSD (2)<br>**Worse** (12 eff)<br>- Read_rec (2)<br>- Phono (1)<br>- Coding (1)<br>- St-Inter (4)<br>- DSF-acc/max (3)<br>- SSB-max (1)	**Freq/Loc** (29 eff)<br>- Beta (24)<br>- TP8-7 (12)<br>- P8-7 (15)<br>- P4-3 (1)<br>**Better** (15 eff)<br>- DSB-acc (1)<br>- SWM Acc (14)<br>**Worse** (14 eff)<br>- ST-Inter (1)<br>- DSB-max (1)<br>- SWM RT (12)

Primary study analyses/outcomes highlighted in green. For “RPA association to: symptoms, temperament, or cognition”, more RPA (i.e., greater rightward parietal asymmetry) is associated with the reported outcomes. **H**= hyperactive; **IA**= inattentive; **Acc**= accuracy; **RTSD**= reaction time standard deviation; **RTSE**=reaction time standard error; Values in parentheses indicate the number of results (i.e., comprised of different frequency bands and/or parietal locations) showing a given effect. **Freq/Loc**= frequency/location; **eff**= effects. See table 2 for description of measures abbreviations.
